# Blocking CXCLs**–**CXCR2 axis in tumor–stromal interactions contributes to survival in a mouse model of pancreatic ductal adenocarcinoma through reduced cell invasion/migration and a shift of immune-inflammatory microenvironment

**DOI:** 10.1038/s41389-018-0117-8

**Published:** 2019-01-18

**Authors:** Makoto Sano, Hideaki Ijichi, Ryota Takahashi, Koji Miyabayashi, Hiroaki Fujiwara, Tomoharu Yamada, Hiroyuki Kato, Takuma Nakatsuka, Yasuo Tanaka, Keisuke Tateishi, Yasuyuki Morishita, Harold L. Moses, Hiroyuki Isayama, Kazuhiko Koike

**Affiliations:** 1 Department of Gastroenterology, Graduate School of Medicine, The University of Tokyo, 7-3-1 Hongo, Bunkyo-ku, Tokyo, 113-8655 Japan; 2 Division of Human Pathology, Department of Pathology and Microbiology, Nihon University School of Medicine, 30-1 Oyaguchikamicho, Itabashi-ku, Tokyo, 173-8610 Japan; 3 Department of Clinical Nutrition Therapy, Graduate School of Medicine, The University of Tokyo, 7-3-1 Hongo, Bunkyo-ku, Tokyo, 113-8655 Japan; 4 Department of Molecular Pathology, Graduate School of Medicine, The University of Tokyo, 7-3-1 Hongo, Bunkyo-ku, Tokyo, 113-0033 Japan; 5 Vanderbilt-Ingram Comprehensive Cancer Center, Vanderbilt University, 691 Preston Building, Nashville, TN 37232 USA; 6 Department of Gastroenterology, Juntendo University School of Medicine, 3-1-3 Hongo, Bunkyo-ku, Tokyo, 113-8431 Japan

## Abstract

Pancreatic ductal adenocarcinoma (PDAC) is characterized by dense stromal reaction (desmoplasia). We have previously reported that mice with conditional *Kras*^G12D^ mutation and knockout of *TGF-β receptor type II* (*Tgfbr2*), PKF mice, develop PDAC with desmoplasia modulated by CXC chemokines that are produced by PDAC cells through tumor–stromal interaction. In this study, we further discovered that PDAC and cancer-associated fibroblast (CAF) accelerated each other’s invasion and migration through the CXC chemokines-receptor (CXCLs–CXCR2) axis. Heterozygous knockout of *Cxcr2* in PKF mice (PKF2h mice) prolonged survival and inhibited both tumor angiogenesis and PDAC microinvasion. Infiltration of neutrophils, myeloid-derived suppressor cells (MDSCs), and arginase-1^+^ M2-like tumor-associated macrophages (TAMs) significantly decreased in the tumors of PKF2h mice, whereas inducible nitric oxide synthase (iNOS)^+^ M1-like TAMs and apoptotic tumor cells markedly increased, which indicated that blockade of the CXCLs–CXCR2 axis resulted in a shift of immune-inflammatory microenvironment. These results suggest that blocking of the CXCLs–CXCR2 axis in tumor–stromal interactions could be a therapeutic approach against PDAC progression.

## Introduction

Pancreatic ductal adenocarcinoma (PDAC), a usually lethal and common type of pancreatic cancer, is the third and fourth leading cause of cancer death in the United States^1^ and Japan^2^. Five-year survival rate is still around 8%, partially attributed to the difficulty of early diagnosis, while postsurgical 5-year survival is still around 20%. The poor prognosis of PDAC is due to aggressive malignant potentials including invasive and metastatic activity^3,4^.

Progression of the PDAC is thought to result in the accumulation of specific genetic alterations through premalignant pancreatic intraepithelial neoplasia (PanIN) and the invasive stage^5^. An activating point mutation of the *KRAS* proto-oncogene is observed in more than 95% of PDAC cases, whereas inactivation of tumor suppressor genes, including *CDKN2A*, *TP53*, and *SMAD4/DPC4*, have been shown to increase in frequency with progression of PanIN to PDAC^6^. Meanwhile, mutation of *TGFBR2* (*TGF-β receptor type II*), a tumor suppressor gene, is less frequently detected in lesions in PanIN/PDAC compared with *SMAD4/DPC4*^7^, both of which function in the TGF-β-SMAD signaling pathway. However, decrease of TGFBR2 expression is observed in nearly half of the PDAC cases^8,9^, indicating that inactivation of TGF-β-SMAD signaling is important in PanINs/PDAC progression.

We previously reported a genetically engineered mouse model with pancreas epithelium-specific activation of *Kras*^*G12D*^ and knockout of *Tgfbr2* (*Ptf1a*^*cre/+*^*;LSL-Kras*^*G12D/+*^*;Tgfbr2*^*flox/flox*^) (called PKF mice), which develops PDAC with abundant stromal reaction (desmoplasia)^10,11^. Indeed, we found that PDAC cells in PKF mice secrete several CXC chemokines (Cxcl 1, 2, 5, and 16) into the tumor stroma and promote desmoplastic reactions and tumor angiogenesis. Inhibition of CXC chemokine receptor type 2 (CXCR2), a G-protein-coupled receptor, blocked the tumor angiogenesis via downregulation of connective tissue growth factor (Ctgf) expression in the tumor-associated fibroblasts (TAFs), leading to an anti-tumor effect and significant survival extension^11^. However, TAFs used in the study were derived from mPanIN tissues and a whole image of the stromal response to the tumor other than Ctgf upregulation in TAFs still remains unclear.

CXC chemokines and CXCR2 were originally considered to contribute to neutrophil chemotaxis in response to tissue injury and infections^12–15^. Recent studies indicated that infiltration of neutrophils and myeloid-derived suppressor cells (MDSCs) through the CXC ligands (CXCLs)–CXCR2 signaling also contribute to tumor progression in various cancers including PDAC^16,17^. Indeed, mice with global knockout of *Cxcr2* demonstrated significant reduction of MPO^+^ neutrophils and Cd11b^+^Ly-6G^+^ MDSCs in *Kras*^*G12D*^-induced PDAC^18^. In addition, the CXCLs–CXCR2 axis can also promote angiogenesis directly through endothelial proliferation and vascular tube formation^19,20^. Thus, the CXCLs–CXCR2 axis is highly involved in PDAC progression, especially in tumor–stromal interactions and the tumor microenvironment.

In the present study, we separated cancer-associated fibroblasts (CAFs) from PKF PDAC tissues and demonstrated upregulation of CXCR2 ligands in the CAFs stimulated by conditioned media of the PDAC cells. In addition, we found that the CXCLs–CXCR2 axis plays important roles in migration or invasion of PDAC cells as well as CAFs in vitro and in vivo. Moreover, we demonstrated that blocking of CXCLs–CXCR2 axis in vivo induces tumor cell apoptosis with a shift of infiltrated immune-inflammatory cells in the PDAC lesions, as evidenced by reduced infiltration of neutrophils/MDSCs and arginase-1-positive macrophages, and increased inducible nitric oxide synthase (iNOS)-positive macrophages, leading to significant survival extension of PDAC-bearing mice. Thus, CXCLs–CXCR2 axis could be one of the potent therapeutic targets for PDAC.

## Results

### PDAC cells and CAFs mutually promote invasion/migration by activating the CXCLs–CXCR2 axis

**Fig. 1:**
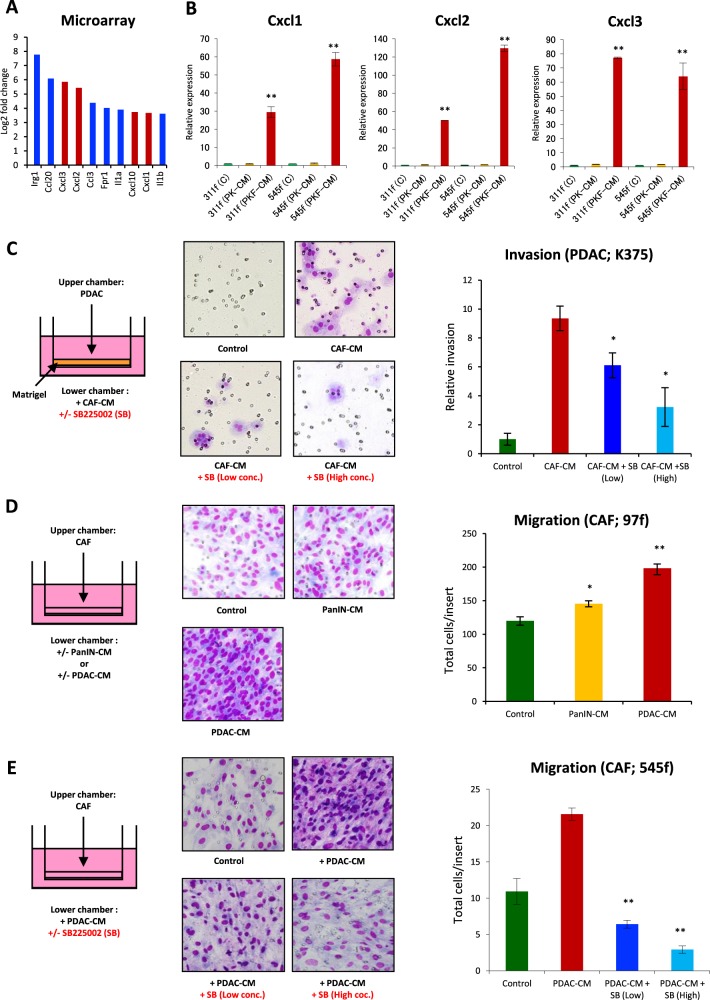
Interactions of PDAC cells and cancer-associated fibroblasts (CAFs) mediated by CXCRLs–CXCR2 signaling pathway. **a** Top 10 upregulated genes in CAFs after stimulation with conditioned medium (CM) of PDAC cell lines derived from PKF (*Ptf1a*^*cre/+*^;*LSL-Kras*^*G12D/+*^;*Tgfbr2*^*flox/flox*^;*Cxcr2*^*+/+*^) mice in the microarray analysis. *Cxcl* genes are red-colored. **b** qRT-PCR analysis in CAFs (311f, 545f) after addition of CM from mPanIN cells (PK-CM) or PDAC cells (PKF-CM). Data are means ± standard error (SE). **p < 0.01 compared to the control (C) and (PK-CM). **c** Invasive activity of PDAC with CM of CAFs with/without CXCR2 inhibitor SB225002. Data are means ± SE. *p < 0.05 compared to the CAF-CM. **d** Migration of CAFs with CM of mPanIN or PDAC. Data are means ± SE. *p < 0.05, **p < 0.01 compared to the control. **e** Inhibition of CAF migration by CXCR2 inhibitor SB225002. Data are means ± SE. **p < 0.01 compared to the PDAC-CM

In this study, we analyzed in detail the response of CAFs to PDAC cells in the tumor–stromal interaction. Characterization of PDAC cells and CAFs was previously reported^11^, whereas additional CAFs obtained from the PDAC tissues of PKF mice were validated. No recombination of the *LSL-Kras*^*G12D*^ and *Tgfbr2*^*flox*^ alleles in the CAFs was detected by polymerase chain reaction (PCR), since the recombination was driven by pancreas epithelium-specific *Ptf1a*^*Cre*^. Almost all the CAFs were spindle shaped and double positive for the expression of α-smooth muscle actin (α-SMA) and fibroblast specific protein 1 (FSP1) (Supplementary Fig. 1). Changes in the gene expression profile in the CAFs after stimulation with conditioned media (CM) of PDAC cells was analyzed using gene-expression microarray, which identified 580 upregulated genes with an adjusted *P* value of 0.05 and greater than twofold change in expression between the groups. Of the 580 genes, major genes are shown in Fig. 1a, Supplementary Fig. 2 and Supplementary Table 1. Interestingly, *Cxc* chemokines including *Cxcl1, Cxcl2*, and *Cxcl3*, which are ligands of CXCR2, were upregulated in CAFs after stimulation by PDAC–CM (Fig. 1a). In addition, the stimulated CAFs synthesized other chemokines such as *Ccl2, Ccl3*, *Ccl6*, *Ccl7*, *Ccl9*, and *Ccl20*, as well as the inflammatory cytokines *IL-1β*, *IL-1β*, and *TNF* (Supplementary Fig. 2 and Supplementary Table 1). Meanwhile, upregulation of *Cxcl1*, *Cxcl2*, *and Cxcl3* in CAFs treated with PDAC–CM was confirmed by quantitative reverse transcription PCR (qRT-PCR) (Fig. 1b and Supplementary Fig. 3). These results indicate that the CAFs produce the same CXCLs that PDAC cells are secreting as a response to stimuli from PDAC cells in the tumor–stromal interaction.

Next, we performed transwell invasion/migration assays to examine the effect of tumor–stromal interactions on the PDAC cells and CAFs. The invasion of PDAC cells was significantly enhanced by addition of CAF-CM (Fig. 1c), whereas the invasion activity was reduced in a dose-dependent manner by CXCR2-specific inhibitor SB225002 (Fig. 1c). Interestingly, migration activity of CAFs was increased by mPanIN cell–CM and was further enhanced by PDAC cell–CM compared with that with mPanIN–CM (Fig. 1d). The migration of CAFs was also decreased by CXCR2 inhibitor SB225002 in a dose-dependent manner (Fig. 1e). Meanwhile, proliferative activity of PDAC cells and CAFs was not affected by CXCR2 inhibitor (Supplementary Fig. 4). These results suggest that PDAC cells promote the migration of CAFs, and in turn, CAFs activate the invasion of PDAC cells, both by secreting the same CXCLs into the tumor microenvironment and activating the CXCLs–CXCR2 axis, without directly affecting cell proliferation. This bidirectional chemokine signaling might be highly involved in the invasion and metastasis of PDAC, which might explain the underlying mechanisms of the extended survival of PDAC-bearing mice with CXCR2 inhibitor treatment, which we have previously reported^11^.

### Heterozygous knockout of Cxcr2 significantly extend the survival of PDAC mice, which is associated with inhibition of microvessel invasion

**Fig. 2:**
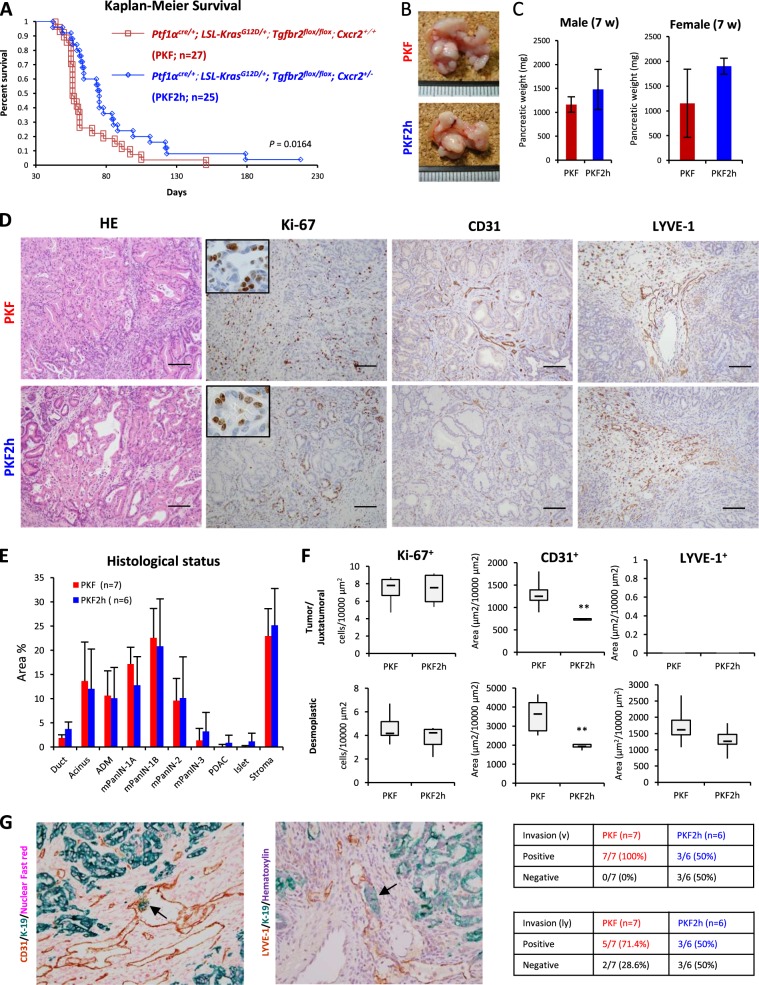
Heterozygous knockout of *Cxcr2* is associated with better outcome of PDAC mice with inhibition of PDAC vessel invasion. **a** Kaplan–Meier survival analysis of PKF (*n* = 27) and PKF2h (*n* = 25; *Ptf1a*^*cre/+*^;*LSL-Kras*^*G12D/+*^;*Tgfbr2*^*flox/flox*^;*Cxcr2*^*hetero+/-*^) mice. *P* = 0.0164 by log-rank test. **b** Gross appearance of pancreatic tumor in PKF and PKF2h mice (7 weeks old (wo)). **c** Pancreatic tumor weight (7 wo). Data are medians ± standard deviation (SD). **d** Histopathological findings including staining for Ki67, CD31, and LYVE-1 in pancreatic tumor in 7-wo PKF (*n* = 7) and PKF2h mice (*n* = 6). Scale bars, 100 μm. Insets: 400× magnification. **e** Histogram of histopathological status of the pancreatic tumor (7 wo). ADM acinar ductal metaplasia. **f** Count of stained cells in the tumor ductal lumen, juxtatumoral, or dense interlobular stroma with desmoplastic reaction (desmoplastic stroma). Data are medians ± SD. *p < 0.05; **p < 0.01. **g** Microscopic invasion of PDAC (arrows) into veins or lymph vessels analyzed by double staining with epithelial marker K-19 and CD31 or LYVE-1

These results suggested importance of the CXCLs–CXCR2 axis in PDAC progression and prompted us to knock out *Cxcr2* in the PKF mice (*Ptf1a*^*cre/+*^;*LSL-Kras*^*G12D/+*^;*Tgfbr2*^*flox/flox*^;*Cxcr2*^*−/−*^) to investigate whether we could dramatically prevent PDAC formation or progression. However, progeny of homozygous Cxcr2 knockout could not be obtained. Although homozygous Cxcr2 knockout was achieved in other PDAC models^18^, it might be due to the different *Cxcr2*^*−/−*^ mouse strain used. Thus, we used heterozygous knockout of *Cxcr2* in the context of PKF (*Ptf1a*^*cre/+*^;*LSL-Kras*^*G12D/+*^;*Tgfbr2*^*flox/flox*^;*Cxcr2*^*+/−*^) (PKF2h). Even with the heterozygous knockout of *Cxcr2*, survival of PKF2h mice was significantly extended compared to that of PKF mice based on Kaplan–Meier analysis (median survival 75 days vs. 56 days, *p* = 0.0164) (Fig. 2a). Gender bias was not detected in the survival ratio, both in the PKF and PKF2h mice.

However, heterozygous knockout of *Cxcr2* did not prevent PDAC formation, and there was no significant difference in gross appearance of pancreatic tumor as well as tumor weight between PKF and PKF2h mice (Fig. 2b, c). Indeed, no statistical difference of histological status, including premalignant mPanINs and PDAC, was found between PKF and PKF2h mice (Fig. 2d, e). The observed number of Ki-67-positive proliferating cells was similar in the tumor/juxtatumoral stroma and desmoplastic stroma (Fig. 2d, f), also indicating that heterozygous knockout of *Cxcr2* in vivo was not directly correlated to proliferation of PDAC.

Next, we performed staining for CD31 and LYVE-1 to study tumor invasion into the vein and lymph vessels, respectively (Fig. 2g), because invasion of PDAC cells was inhibited by CXCR2 inhibitor in vitro. Similar to the invasion assay, PDAC microinvasion into CD31^+^ tumor blood vessels was significantly reduced in PKF2h mice (3/6; 50%) compared to PKF mice (7/7; 100%). In addition, microinvasion into LYVE-1^+^ lymph vessels was decreased in PKF2h mice (3/6; 50%) compared to PKF mice (5/7; 71.4%). Interestingly, CD31^+^ tumor microvessel density significantly decreased in both the pancreatic juxtatumoral and desmoplastic stroma of PKF2h mice (Fig. 2d, f). Therefore, it is considered that heterozygous knockout of *Cxcr2* in PDAC cells, CAFs and endothelial cells, in which CXCR2 activation by PDAC–CAF interactions is interrupted, leads to inhibition of PDAC vessel invasion. Meanwhile, heterozygous *Cxcr2* knockout in endothelial cells of blood vessels may block tumor angiogenesis during the PDAC progression in PKF2h mice.

### Antitumor effect is induced by increase of antitumor iNOS^+^ macrophages in heterozygous *Cxcr2* knockout PDAC mice

**Fig. 3:**
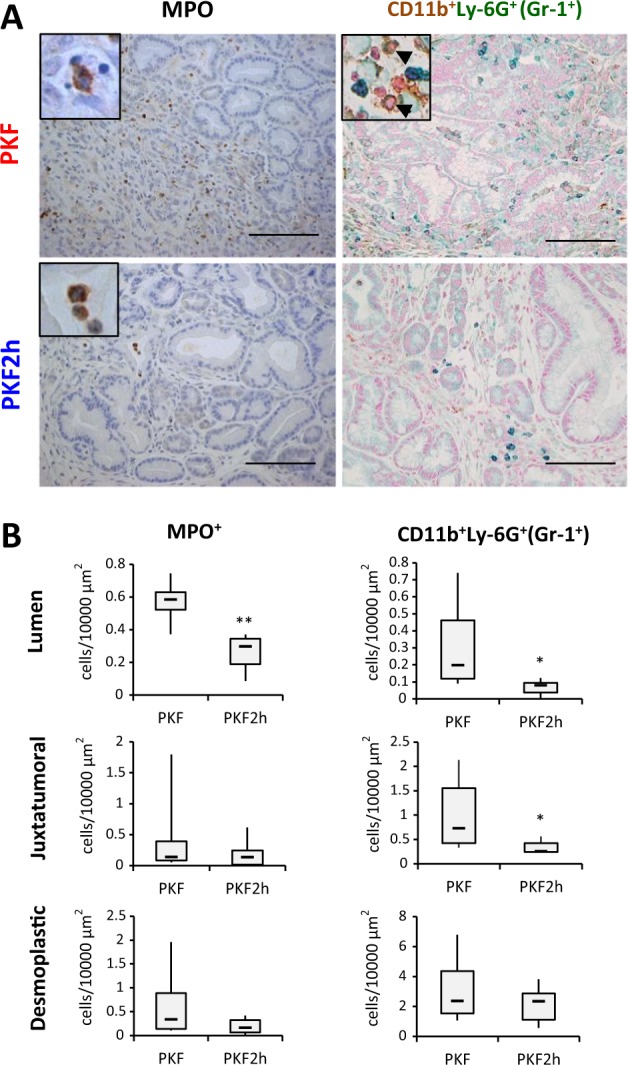
Heterozygous knockout of *Cxcr2* inhibits infiltration of MPO^+^ and CD11b^+^/Ly-6G^+^ granulocytes in pancreatic tumor. **a** Immunostaining for MPO and double staining for CD11b and Ly-6G in pancreatic tumor of PKF and PKF2h mice (7 wo). Scale bars, 100 μm. Insets: 400× magnification. **b** Quantification of MPO^+^ and CD11b^+^/Ly-6G^+^ cells. Data are medians ± SD. *p < 0.05; **p < 0.01

In other *Cxcr2*-knockout mouse models, significant decrease of neutrophil infiltration into the local area including dermatitis and PDAC has been reported^18,21,22^. We thus investigated inflammatory cell infiltration in the pancreatic tumor of PKF and PKF2h mice. Similar to the previous reports, decrease of myeloperoxidase^+^ (MPO^+^) neutrophil infiltration was observed in the pancreatic tumor area in PKF2h mice (Fig. 3a, b). We performed double staining with CD11b and Ly-6G, because infiltration of MDSCs is known to be important in PDAC progression^23^, and observed that CD11b^+^Ly-6G^+^ MDSCs significantly decreased in the tumor of PKF2h mice (Fig. 3a, b).

**Fig. 4:**
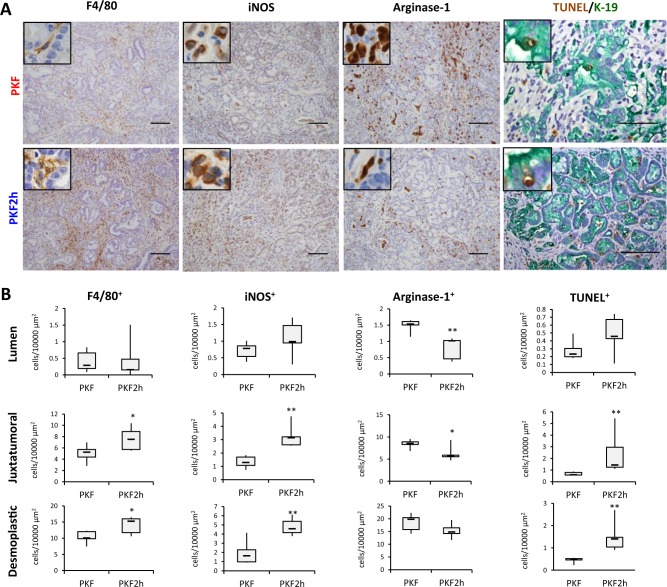
Inhibition of *Cxcr2* induces iNOS^+^ macrophage infiltration and TUNEL^+^ apoptotic cells. **a** Detection of macrophage markers (F4/80, iNOS, and arginase-1) by immunohistochemistry or apoptotic cells using TUNEL (brown) and K-19 double staining in PKF and PKF2h mice (7 wo). Scale bars, 100 μm. Insets: 400× magnification. **b** Quantification of F4/80^+^, iNOS^+^ or arginase-1^+^ macrophages and TUNEL^+^ apoptotic cells in the tumor location. Data are medians ± SD. *p < 0.05; **p < 0.01

Numerous F4/80^+^ macrophages also infiltrated in the tumor stroma with dense desmoplastic reaction in both PKF and PKF2h mice (Fig. 4a, b). Inflammatory macrophages can be classified into two types: pro-inflammatory M1 and anti-inflammatory M2 macrophages^24^. M1 macrophages express iNOS, which metabolizes arginine to NO and plays a role in antitumor effect, whereas M2 macrophages express arginase, which hydrolyzes arginine to ornithine and urea, and has anti-inflammatory and tissue repair effects^24^. Thus, we next performed staining for iNOS and arginase-1 to functionally determine the phenotype of infiltrated macrophages as M1 or M2. Interestingly, iNOS^+^ M1-like macrophages significantly increased in the tumor stroma of PKF2h mice, whereas arginase-1^+^ M2-like macrophages tended to decrease (Fig. 4a, b). TUNEL^+^ apoptotic cells significantly increased in PKF2h mice (Fig. 4a, b), which was consistent with the previous report that classical iNOS^+^ M1-like tumor-associated macrophages (TAMs) possess anti-tumor effects by secreting cytoplasmic NO and inducing apoptosis^25^. In the TUNEL/K-19 double staining, we observed that TUNEL^+^ cells in the juxtatumoral area were also K-19^+^ tumor cells (Fig. 4a), which indicated that apoptotic tumor cells increased in PKF2h compared with PKF mice. We also observed that TUNEL^+^/K-19^−^ apoptotic stromal cells increased in the desmoplastic area in PKF2h mice.

**Fig. 5:**
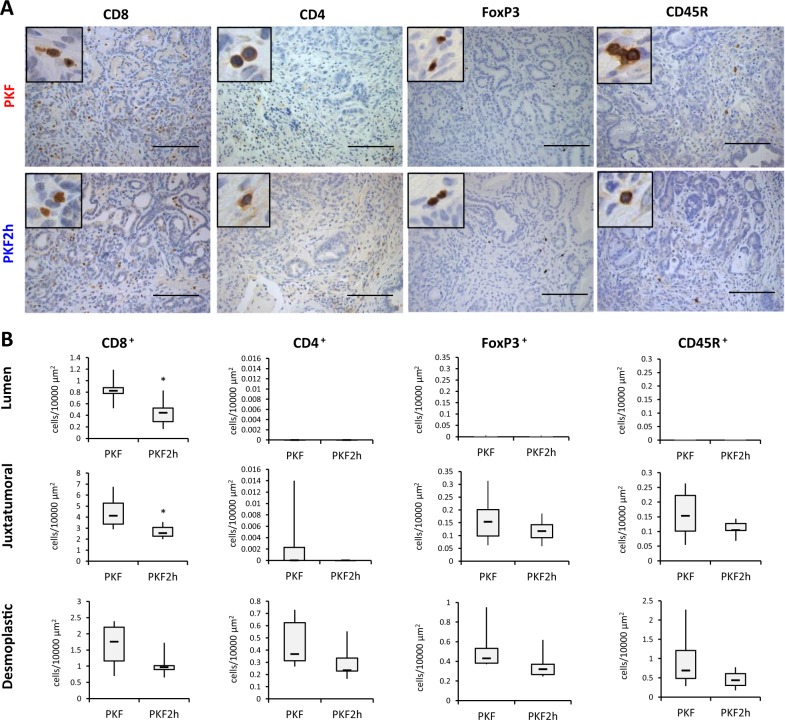
*Cxcr2*-heterozygous knockout inhibits infiltration of CD8^+^ and FoxP3^+^ lymphocytes in the tumor lesion. **a** Immunohistochemical analyses for lymphocytic markers in PKF and PKF2h. Scale bars, 100 μm. Insets: 400× magnification. **b** Quantification of stained lymphocytes in pancreatic tumors. Data are medians ± SD. *p < 0.05; **p < 0.01

Infiltration of lymphocytes was also analyzed in PKF and PKF2h mice, which revealed that infiltration of cytotoxic CD8^+^ T cells and regulatory T cell-specific FoxP3^+^ cells decreased in the pancreatic tumor nest in PKF2h mice, whereas no significant difference of CD4^+^ suppressor T cells and CD45R^+^ B lymphocytes was detected between PKF and PKF2h mice (Fig. 5a, b). These results suggest that infiltration of iNOS^+^ M1-like TAMs, but not cytotoxic CD8^+^ lymphocytes, could have an important role in the anti-tumor effect on PDAC in PKF2h mice. Reduced infiltration of MPO^+^ neutrophils and MDSCs might also be involved in the inhibited PDAC progression in PKF2h mice.

## Discussion

**Fig. 6:**
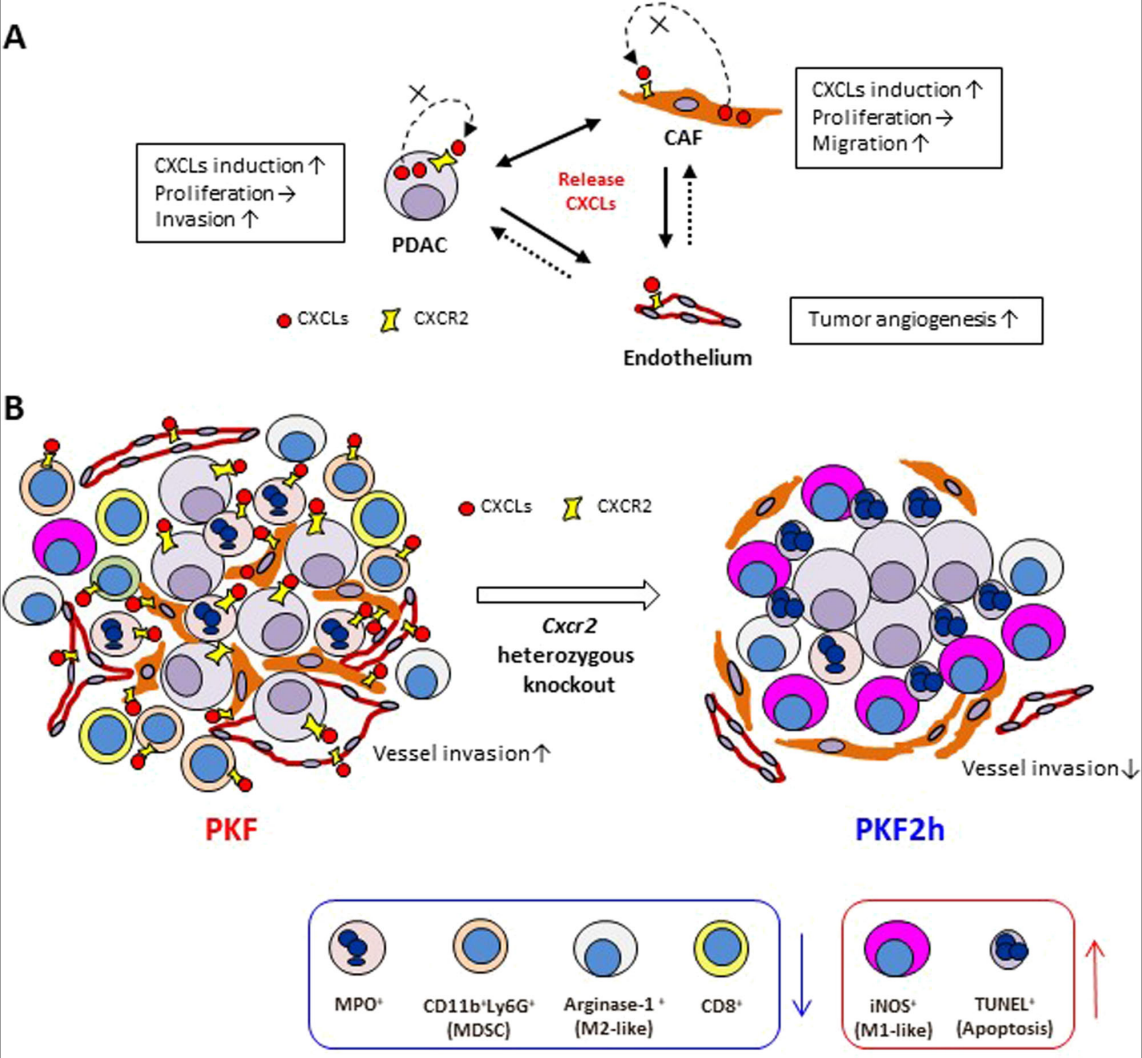
Hypothetical mechanism of PDAC progression/inhibition according to the *Cxcr2*-heterozygous knockout PKF2h model. **a** Secretion of CXCLs from PanIN/PDAC and CAF stimulates CXCL expression and migration/invasion activity via CXCR2. Induction of CXCLs in these cells also promotes tumor angiogenesis. Dotted line represents unclear functions. **b** In the *Cxcr2*-heterozygous knockout pancreatic tumor of PKF2h mice, recruitment of MPO^+^ neutrophils, CD11b^+^Ly-6G^+^ MDSCs and arginase-1^+^ M2 macrophages is inhibited compared to PKF mice. In contrast, iNOS^+^ M1 macrophages and TUNEL^+^ apoptotic cells significantly increase in the pancreatic tumor. Micro-vessel density of CD31^+^ vessels is decreased and PDAC microinvasion into the vein and lymph vessels is inhibited in PKF2h pancreatic tumor

In this study, we explored stromal–tumor interactions in PDAC to elucidate how CAFs respond to the tumor cells. By screening upregulated factors in mouse CAFs after stimulation with PDAC cell conditioned medium, we determined that Cxcl1–3 were upregulated in CAFs, some of which were also highly produced by PKF-derived PDAC cells^11^. In human PDAC, expression of CXCL1 and -5 is upregulated compared with that in normal pancreatic duct, whereas high levels of CXCL1, -5, and -8 are significantly detected in the pancreatic fluid of PDAC patients^19,26^, which may also be partially derived from CAFs. Previously, we focused on Ctgf, which was induced in mPanIN-derived TAFs by PDAC stimuli^11^; however, we found in this study that upregulation of the Cxcls was much higher than that of Ctgf in the CAFs derived from the PDAC. In addition, this study revealed that the activated CXCLs–CXCR2 axis promoted invasion of PDAC cells and migration of CAFs. The PDAC cells and CAFs might attract each other by secreting the CXCLs and invade together; this may contribute to the PDAC invasion and desmoplasia formation, and might support the idea that invasion and metastasis of PDAC cells are accompanied and/or directed by CAFs^27^. A scheme of suggested tumor-promoting tumor–stromal interactions is shown in Fig. 6a.

This study suggests that heterozygous *Cxcr2* knockout causes a shift of immune–inflammatory tumor microenvironment as shown in Fig. 6b. In the heterozygous *Cxcr2* knockout PKF2h mice, MPO^+^ neutrophils and CD11b^+^Ly-6G^+^ MDSCs significantly decreased in the tumor nest. Another study also showed significant reduction of neutrophil and MDSC infiltration in the PDAC mice with global knockout of *Cxcr2*^18^. In contrast, pancreas epithelial-specific loss of *Cxcr2* induced recruitment of neutrophils and MDSCs into the tumor nest and enhanced PDAC metastasis^18^. Therefore, the *Cxcr2* knockout in the stromal components of PDAC is contributing to the inhibition of neutrophil and MDSC recruitment, which might be one of the antitumor mechanisms.

Infiltration of F4/80^+^ macrophages is closely related to *Kras*^*G12D*^-induced acinar ductal metaplasia, mPanIN and PDAC development^28^. In an experimental inflammatory model in *Cxcr2*-null mice, marked reduction of neutrophil infiltration was observed in the cutaneous inflammation, while Mac2^+^ macrophages significantly increased^22^. Likewise, in contrast to neutrophils, F4/80^+^ TAMs significantly infiltrated into the tumor lesions in our *Cxcr2*-heterozygous knockout mice as well as in the other PDAC model with *Cxcr2*-knockout^18^. In general, macrophages infiltrate at the chronic inflammatory stage after acute inflammation with neutrophil infiltration. Thus, neutrophil reduction could induce F4/80^+^ TAM infiltration in the tumor nest in an innate manner. Presence of TAMs has been recognized as a poor prognostic marker in cancers including PDAC^29^. TAMs enhance invasion/metastasis and neovascularization of PDAC and inhibit the adaptive tumor-specific immune response by secreting proteases, chemokines and angiogenic factors^29^. In particular, M2-like TAMs are increased in the tumor lesion and associated with tumor progression and poor prognosis^29^. We further evaluated the functional markers of macrophages and found that infiltration of arginase-1^+^ M2-like TAMs decreased in the mPanIN/PDAC lesions, whereas iNOS^+^ M1-like TAMs increased in the tumor area, resulting in a significant induction of apoptosis of the tumor cells (Fig. 6b). M2-like TAMs are recently known to be transformed into M1 macrophages in pathological environments^30,31^. Although the detailed mechanism behind decreased M2-like TAMs and increased M1-like TAMs in PKF2h mice remains to be elucidated, increase of M1/M2 ratio by *Cxcr2* inhibition would be effective for modulating the immune tumor microenvironment of PDAC.

In contrast with the neutrophil, MDSC and macrophage infiltration, the impact of infiltrated lymphocytes appeared to be small in this study. Another group reported that CD3^+^ T cell infiltration increased in the pancreatic tumor nest in PDAC mice with global knockout of *Cxcr2*^18^. In addition, cytotoxic subpopulations of CD8^+^ T cells were not described and the increased CD3^+^ T cells did not prolong the survival of *Cxcr2* knockout mice significantly; however, cytotoxic activity of the CD3^+^ T cells was suggested after treatment with programmed cell death protein 1 (PD-1) antibody. In our model, tumor-infiltrated cytotoxic CD8^+^ T cells decreased in heterozygous *Cxcr2* knockout PKF2h mice. Even in PKF mice with *Cxcr2*-wild type, only a few CD8^+^ T cells were observed in the tumor lesions. The number of infiltrated CD4^+^ helper T cells, FoxP3^+^ regulatory T cells, and CD45R^+^ B lymphocytes was also small in the tumor lesions of PKF and PKF2h mice.

In this study, heterozygous knockout of *Cxcr2* significantly extended the survival of PDAC mice. Recent studies demonstrated that treatment with CXCR2 inhibitor prolonged the survival of two different PDAC models, *Kras*^*G12D*^ + *Tgfbr2* knockout (PKF) and *Kras*^*G12D*^ + Tp53 inactivation (KPC)^11,18^, which is consistent with this study. However, global homozygous knockout of *Cxcr2* in the KPC mice did not extend the survival^18^. Thus, progression of PDAC with *Kras*^*G12D*^ + *Tgfbr2* knockout might be more dependent on the CXCLs–CXCR2 axis than the PDAC with *Kras*^*G12D*^ + Tp53 inactivation. Our previous observation and current study suggested some of the underlying mechanisms in the *Kras*^*G12D*^ + *Tgfbr2* knockout: the Cxc chemokines expression in the pancreatic epithelial cells was highly correlated with TGF-β signaling, upregulated in the Tgfbr2-knockout cells and downregulated by adding TGF-β in the Tgfbr2-intact cells^11^, the Cxcls expression in the CAFs was induced by the PDAC cells, PDAC cell invasion and CAF migration were induced by each other in a Cxcr2-dependent manner. Although further investigations are necessary to elucidate the detailed mechanisms of the CXCLs–CXCR2 axis dependency of PDAC progression, blocking CXCLs–CXCR2 axis in the crosstalk between tumor and stromal cells could be a promising therapeutic strategy for PDAC.

## Materials and methods

### Cell lines and reagent

Establishment and characterization of cell lines including *Ptf1a*^*cre/+*^;*LSL-Kras*^*G12D/+*^;*Tgfbr2*^*flox/flox*^ mouse (PKF)-derived pancreatic ductal adenocarcinoma (PDAC; K375 and K399), *Ptf1a*^*cre/+*^;*LSL-Kras*^*G12D/+*^ mouse (PK)-derived mPanIN tissues (K512 and K518) and cancer-associated fibroblast (CAF; 97f) have been described^10,11,32^. CAFs (311f, 545f) were also established from primary PDAC sites in PKF mice and were validated by recombination PCR of *LSL-Kras*^*G12D*^ and *Tgfbr2*^*flox*^ alleles^10^ and immunofluorescence with α-SMA and FSP1 (Supplementary Fig 1). CXCR2 inhibitor SB225002 was obtained from Calbiochem.

### Gene-expression profiling microarray

After stimulation by conditioned medium of mixed PDAC cell lines (K375 and K399), RNAs of mixed CAF cell lines (97f, 311f, and 545f) were extracted using Nucleospin RNA II kit (TaKaRa) and treated with RQ1 DNase (Promega), then the RNA samples were labeled using the 3′ IVT Express Kit (Affymetrix, 901,228) and subjected to gene expression profiling using GeneChip Mouse Genome 430 2.0 arrays (Affymetrix, 900,495). Raw data is available in Figshare repository (https://figshare.com).

### qRT-PCR

cDNA was generated from 0.5 μg of total RNA using ImProm-II Reverse Transcription system (Promega). qRT-PCR was performed using FastStart Universal SYBR Green Mastermix (Roche) and StepOnePlus realtime PCR system (Applied Biosystems) according to the manufacturers’ instructions. The amount of PCR product was normalized against GAPDH as an internal control. Experiments were repeated twice in duplicate.

Primer pairs used are shown in Supplementary Table 2.

### Matrigel invasion assay

Matrigel invasion assay was performed using BD BioCoat Matrigel Invasion Chamber (BD Biosciences). PDAC cells (5 × 10^4^) were plated into Matrigel-coated invasion chamber inserts and incubated with 20% FBS-containing culture media for 48 h, with or without CAF-derived conditioned media in the lower chamber. The invaded cells were counted using Diff-Quick stain (Sysmex) as described previously^33^. To determine the effect of CXCLs-CXCR2 axis, 0.4 or 4 μM SB225002 or DMSO (control) was added into the lower chamber containing CAF-derived conditioned media. Experiments were repeated three times in triplicate.

### Migration assay

CAFs (2 × 10^5^) were plated into the upper chamber inserts (BD Biosciences) and incubated with 20% FBS-contained culture media for 24 h, with or without mPanIN- or PDAC-derived conditioned media in the lower chamber. The migrated cells were counted using Diff-Quick stain. To determine the effect of CXCLs–CXCR2 axis, 0.4 or 4 μM SB225002 or DMSO (control) was added into the lower chamber containing PDAC-derived conditioned media. Experiments were repeated three times in triplicate.

### Mice

Generation of *Ptf1a*^*cre/+*^;*LSL-Kras*^*G12D/+*^;*Tgfbr2*^*flox/flox*^;*Cxcr2*^*+/+*^ (PKF) mice and the genotyping have been described previously^10,11^. *Ptf1a*^*cre/+*^;*LSL-Kras*^*G12D/+*^;*Tgfbr2*^*flox/flox*^;*Cxcr2*^*+/-*^ (PKF2h) mice were generated by crossing PKF mice with *Cxcr2*^*+/−*^ mice obtained from Jackson Laboratories^34^. Background of all the mice used in this study was C57BL/6, after repeated backcrossing. All animals were kept in specific pathogen-free housing with abundant food and water under guidelines approved by the ethics committee for the Care and Use of Laboratory Animals of the University of Tokyo.

### Histology and immunohistochemistry (IHC)

All murine samples were fixed in 4% paraformaldehyde and embedded in paraffin, as described previously^10,11^. Three-micrometre sections were stained with hematoxylin & eosin (HE) and mPanIN/PDAC status were analyzed as described previously^35^. Antibodies used for IHC staining are summarized in Supplementary Table 3. The staining was performed as described previously^11,35^. DAB substrate (DAKO Japan) or HistoGreen/HISTOPRIME^®^ (E109; Linaris-Biologische Produkte) was used for the development of IHC. For quantification of HE and IHC, 7 week old PKF mice (*n* = 7) and PKF2h mice (*n* = 6) were consecutively analyzed by the investigator blinded to the group allocation. Four random files from each sample were analyzed at 200× original magnification and positive cells were counted in the tumor ductal lumen, juxtatumoral, or dense interlobular stroma with desmoplastic reaction (desmoplastic stroma).

### Statistics

All data of in vitro are indicated as means ± standard error (SE), while in vivo data are indicated as means ± standard deviation. Statcel software version 4 (OMS Ltd.) and JMP statistical software (SAS Institute, Inc.) were used for the statistical calculations^36^. Two-sided student’s *t* test was used for the in vitro invasion/migration, proliferation and qRT-PCR analyses. Two-sided Mann–Whitney’s *U* test was used to determine the significant difference in histopathological analyses. For comparison of survival in Kaplan–Meier analyses, littermates of PKF (*n* = 27) and PKF2h (*n* = 25) were consecutively employed and log-rank test was used for univariate survival analyses. *P* < 0.05 was the threshold for statistical significance.

## Supplementary information

Supplemental Material

Figure S1

Figure S2

Figure S3

Figure S4

Table S1

Table S2

Table S3
