# Supplementary material for: Blocking CXCLs–CXCR2 axis in tumor–stromal interactions contributes to survival in a mouse model of pancreatic ductal adenocarcinoma through reduced cell invasion/migration and a shift of immune-inflammatory microenvironment
Source: Oncogenesis. 2019 Jan 18;8(2):8. doi: 10.1038/s41389-018-0117-8 (PMC6338726; doi:10.1038/s41389-018-0117-8)
Supplement: Supplementary file 1 — Supplemental Material [file 41389_2018_117_MOESM1_ESM.docx]

**Supplemental Methods**

**Cell Proliferation Assay**

PDAC cells (4 × 10^3^) were plated into collagen-coated 96-well plates and incubated with 20% FBS-containing culture media with or without normal fibroblast (NF)- or CAF-derived conditioned media (CM) for 48 h. CAFs (4 × 10^3^) were also plated into collagen-coated 96-well plates and incubated with 20% FBS- containing culture media with or without mPanIN- or PDAC-derived CM for 48 h. In a series of wells with CAF- or PDAC-derived CM, CXCR2 inhibitor SB225002 was also added at 0.4 or 4 μM. Viable cells were quantified using Cell Counting Kit-8 (Dojindo Molecular technologies, inc.) at 0, 24 and 48 h ^11^. Experiments were repeated twice in triplicate.

**Immunofluorescence**

CAFs were subjected to immunofluorescence to examine α-SMA and FSP1 expression as described previously ^40^. Briefly, seeded cells were fixed with 2% paraformaldehyde, permeabilized with 0.25% TritonX-100, and incubated with primary antibodies. Alexa Fluor 488 goat anti-mouse IgG and Alexa Fluor 555 goat anti-rabbit IgG were used as secondary antibodies (Invitrogen) and Hoechst was used for nuclear staining. The primary antibodies used are: α-SMA (DAKO, M0851, 1:100 dilution) and FSP1 (Abcam, ab27957, 1:100 dilution).

**Supplemental Figure 1.** Representative images of CAFs separated from the PDAC tissues of PKF mice. Phase contrast image and immunostaining of α-SMA, and FSP1. Hoechst: nuclear staining. 3T3 cells are used as a control.

**Supplemental Figure 2.** Representative gene expression profiles in CAFs after stimulation with medium conditioned by PDAC cells.

**Supplemental Figure 3.** Relative expression of *Cxcl* chemokines in CAF (97f) with/without conditioned medium of mPanIN (PK) and PDAC (PKF). Data are means ± standard error (SE). **p* < 0.05, ***p* < 0.01 compared to the control (C) and (PK-CM).

**Supplemental Figure 4.** PDAC and CAF proliferation following addition of conditioned media with/without CXCR2 inhibitor. Data are means ± standard error (SE). Cont: control, PK-CM: conditioned medium (CM) of mPanIN (PK) cells, PKF-CM: CM of PDAC (PKF) cells, PKF-CM+SB-L: PKF-CM with low concentration of SB225002 (0.4 μM), PKF-CM+SB-H: PKF-CM with high concentration of SB225002 (4 μM), NF-CM: CM of normal fibroblast (NF), CAF-CM: CM of cancer-associated fibroblast (CAF), CAF-CM+SB-L: CAF-CM with low concentration of SB225002 (0.4 μM), CAF-CM+SB-H: CAF-CM with high concentration of SB225002 (4 μM).
