# Supplementary figures and images for: Blocking CXCLs–CXCR2 axis in tumor–stromal interactions contributes to survival in a mouse model of pancreatic ductal adenocarcinoma through reduced cell invasion/migration and a shift of immune-inflammatory microenvironment
Source: Oncogenesis. 2019 Jan 18;8(2):8. doi: 10.1038/s41389-018-0117-8 (PMC6338726; doi:10.1038/s41389-018-0117-8)

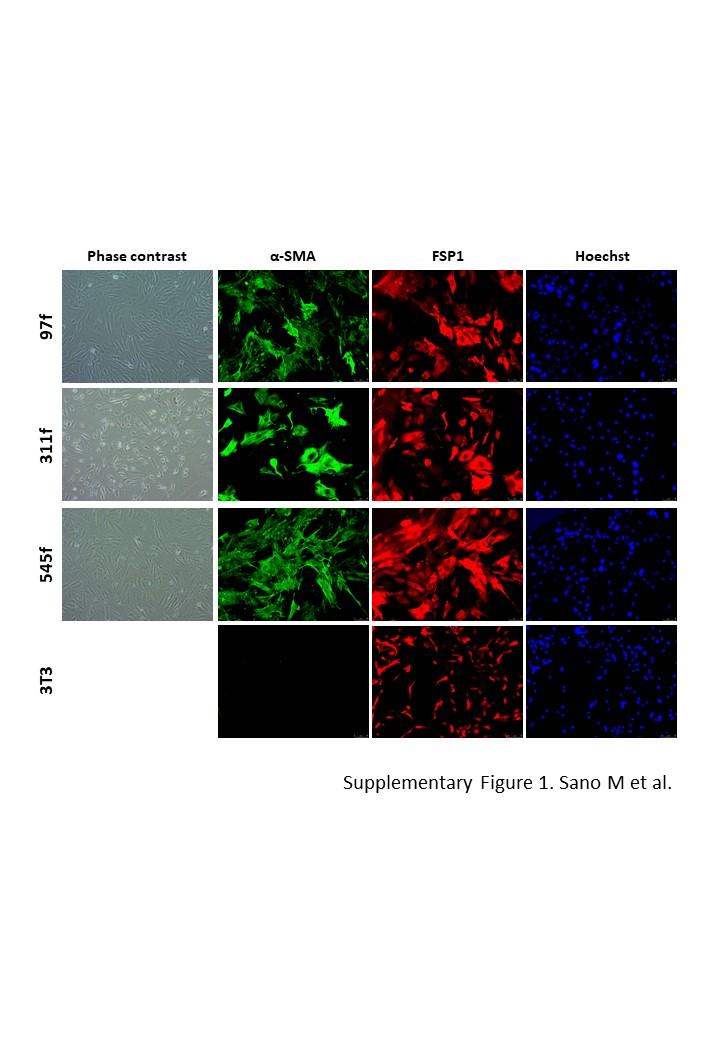

Supplement: Supplementary file 2 — Figure S1 [file 41389_2018_117_MOESM2_ESM.jpg]

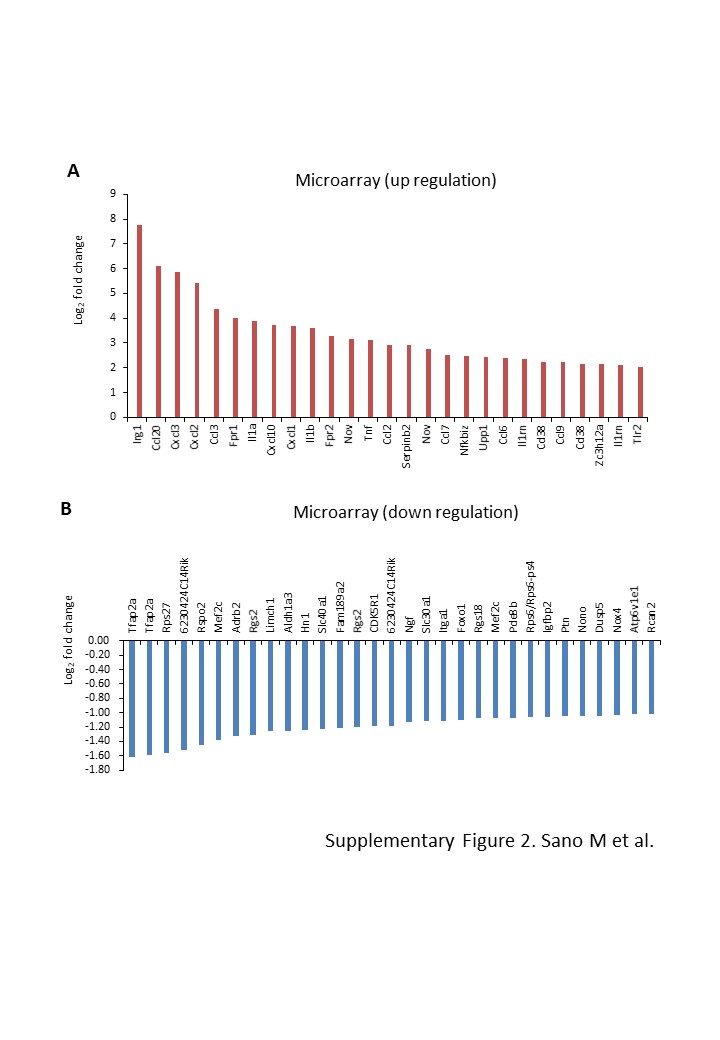

Supplement: Supplementary file 3 — Figure S2 [file 41389_2018_117_MOESM3_ESM.jpg]

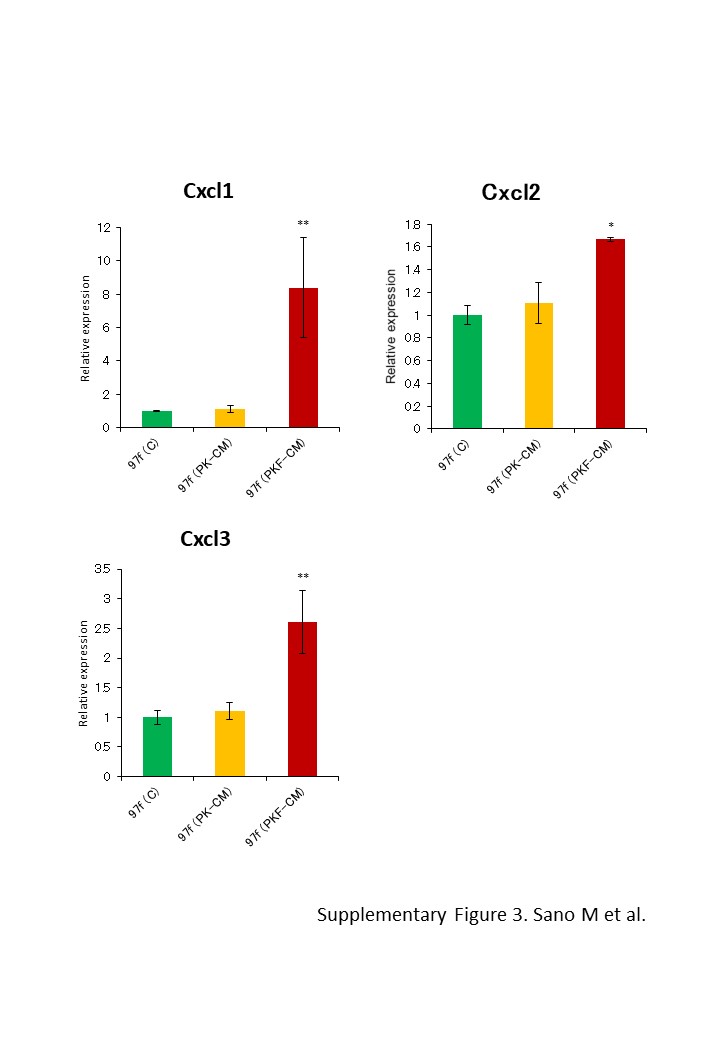

Supplement: Supplementary file 4 — Figure S3 [file 41389_2018_117_MOESM4_ESM.jpg]

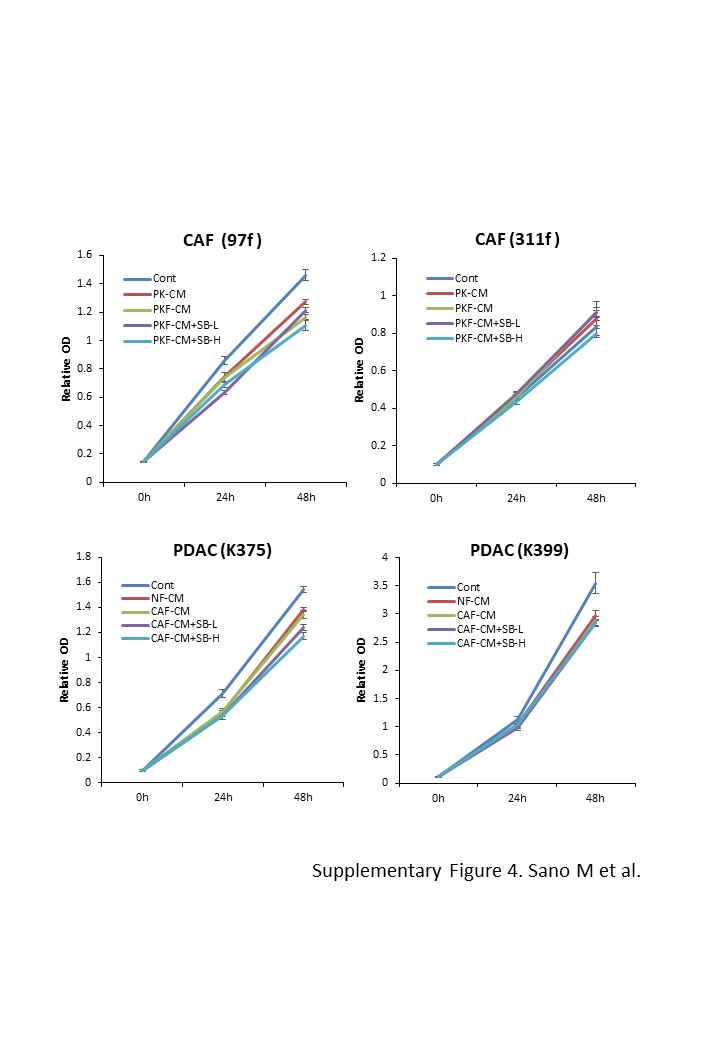

Supplement: Supplementary file 5 — Figure S4 [file 41389_2018_117_MOESM5_ESM.jpg]
